# Silicon Carbide-Based DNA Sensing Technologies

**DOI:** 10.3390/mi14081557

**Published:** 2023-08-04

**Authors:** Abdulla Al Mamun, Mason McGarrity, Jong-Hoon Kim, Feng Zhao

**Affiliations:** School of Engineering and Computer Science, Washington State University, Vancouver, WA 98686, USA

**Keywords:** DNA, SiC, sensor, chemiresistor, label-free, sensitivity

## Abstract

DNA sensing is critical in various applications such as the early diagnosis of diseases and the investigation of forensic evidence, food processing, agriculture, environmental protection, etc. As a wide-bandgap semiconductor with excellent chemical, physical, electrical, and biocompatible properties, silicon carbide (SiC) is a promising material for DNA sensors. In recent years, a variety of SiC-based DNA-sensing technologies have been reported, such as nanoparticles and quantum dots, nanowires, nanopillars, and nanowire-based field-effect-transistors, etc. This article aims to provide a review of SiC-based DNA sensing technologies, their functions, and testing results.

## 1. Introduction

DNA (Deoxyribonucleic Acid) is a biochemical that contains the genetic code and information about the molecule or biological entity [1,2]. Over the last few years, researchers have been highly interested in developing biosensors to selectively detect DNA with high sensitivity for various applications. DNA sensing enables the early diagnosis of fatal diseases, potentially saving millions of lives, and can also be used in the investigation of forensic evidence, food processing, agriculture, environmental protection, and more [3]. All these applications require simple, reliable, rapid, and label-free DNA sensing techniques for high-quality analysis. Semiconductor-based devices for DNA sensing have received considerable attention due to their capability for on-chip, miniaturization, integration, and low-cost manufacturing. Silicon (Si) is the backbone of the semiconductor industry [4] and one of the most commonly used materials for bio-sensing applications [5], mainly because of its electrical properties and well-established micro- and nanofabrication technologies [2]. However, Si can undergo chemical reactions in the body’s environment and is therefore not biocompatible [6,7,8,9] and suffers from instability in long-term uses in biological media and aqueous solutions, resulting in low signal-to-noise ratio and sensor performance issues [2,10,11,12]. Researchers have been exploring other materials for DNA sensing which are biocompatible, chemically inert and robust, and nontoxic to biomedical environments, such as gallium nitride (GaN) [5], carbon nanotubes (CNTs) [13,14,15], graphene [16,17], silicon carbide (SiC) [1,2,10,18,19,20,21,22,23,24,25,26], etc. Among them, SiC is one of the most promising materials for DNA sensing.

SiC is a wide-bandgap semiconductor material that exists in more than 200 polytypes in its single crystalline form, with the 3C, 4H, and 6H polytypes being the most common. “C” refers to cubic crystal structures, “H” refers to hexagonal crystal structures, and the numbers 3, 4, and 6 refer to the stacking sequence—the number of layers of Si and carbon (C) atoms before the atomic arrangement repeats. Depending on the stacking order, the bonding between Si and C atoms in adjacent bi-layer planes is either of a zinc-blende (cubic) or wurtzite (hexagonal) nature. The crystal structure and stacking sequence of 3C-, 4H-, and 6H-SiC are shown in [27]. Table 1 summarizes the material properties of different SiC polytypes.

SiC has excellent electrical properties [28,29,30,31,32,33,34,35] including electron and hole mobility, breakdown electric field, and high conductivity by doping (B, Al, N_2_), etc., making it suitable for bioelectronics and biosensor applications [33]. Another key benefit of SiC is its capacity to grow a thermally stable native silicon dioxide (SiO_2_) layer, which makes it directly compatible with well-established micro- and nanofabrication technologies [24,36] for silicon-based devices and complementary metal-oxide semiconductor (CMOS) applications. The human body contains a variety of salinized and ionic chemicals and proteins, creating a harsh environment that can react with sensing materials or produce a coating on the material surface [34]. The human body’s protection system can easily detect foreign elements and initiate multifarious physical and chemical responses to eliminate or diffuse foreign elements by oxidizing their surface [34]. If the human body cannot eliminate the foreign elements, it will prevent them from accessing the body’s internal environment. Therefore, biosensor materials must have biocompatible properties to minimize undesired reactions between the sensor and the body. SiC is widely recognized for its nontoxicity, biocompatibility, chemical inertness and stability, and mechanical properties such as high Young’s modulus, elasticity, hardness, low friction coefficient, etc. [2,10,22,37,38,39,40], making it compatible with various in vivo and in vitro biomedical applications [34,41] and ideal for long-term implantation in living tissue [33]. Moreover, SiC is transparent at visible wavelengths, has a high refractive index, and absorbs UV light; all these properties make it an excellent choice of material for optoelectronic biosensors [37,42,43,44]. The versatile reactivity of the Si and C terminals at the surface of SiC enables various functionalization strategies to detect DNA, glucose, and many other biomolecules [32].

Recently SiC-based biosensors have been studied to explore their potential for DNA sensing, with six main technologies reported in the literature. These technologies include: (1) aptamer-modified SiC nanoparticles and quantum dots as fluorescent aptasensor [18,25,45], (2) silicon carbide nanoparticle-modified glassy carbon electrode [1], (3) bio-functionalized SiC nanopillar arrays [10,23], (4) bio-functionalized nanowires [19], (5) nanowire-based field effect transistor [2,20,21,23,24,46,47], and (6) nanocrystalline 3C-SiC electrodes [22,41,48,49,50]. This paper presents a concise review of these six SiC-based DNA sensing technologies. The testing results from these technologies, when combined with the advantages enabled by the properties of SiC including its electrical conductivity, thermal and mechanical stability, compatibility with Si CMOS fabrication, nontoxicity, biocompatibility, chemical inertness, surface functionalization capability, etc., testify a promising prospective of SiC for DNA-sensing applications.

## 2. SiC DNA-Sensing Technologies

Nowadays, research communities are highly interested in electrochemical, optical, and electrical-based biosensors for DNA sensing. This section reviews the recent development of SiC DNA sensors and presents our work on label-free DNA sensing with a planar 4H-SiC chemiresistor.

### 2.1. Aptamer-Modified SiC Nanoparticle and Quantum Dot Aptasensors

Luminescence nanocrystal or quantum dots (QDs) are semiconducting nanoparticles that are only a few nanometers in size. They possess the ability to transport electrons, but their electrical and optical properties differ from those of the bulk material [51,52,53]. These nanoparticles emit light of various colors when UV light is incident upon them [51,52]. These semiconducting QDs are highly promising for various biomedical and biosensing applications [53] with advantages [54] such as broad excitation and narrow emission spectrum, resistance to photobleaching, etc. The most commonly used QD materials are cadmium selenide (CdSe), cadmium telluride (CdTe), indium phosphide (InP), and indium arsenide (InAs). However, these QDs are known to exhibit cytotoxicity in living cellular environments [53].

**Figure 1:**
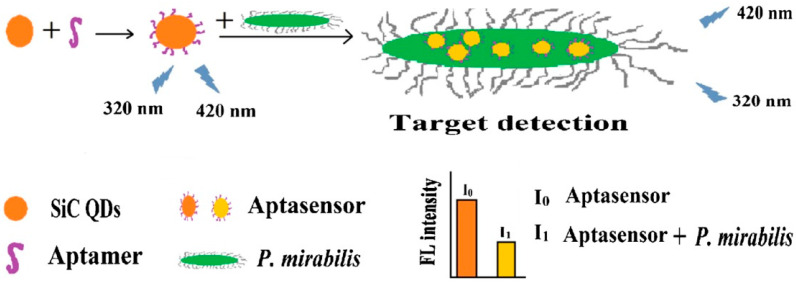
Schematic illustration of *P. mirabilis* detection by fluorescence aptasensor assay. Reprinted with permission from Ref. [25]. 2020, Microchimica Acta.

The investigation of SiC QD-based DNA-sensing technologies has been reported in several studies [18,25,45], One example of a SiC QD-based device for DNA sensing is the fluorescent aptasensor, as reported in [25]. This type of aptasensor utilizes an aptamer with a high affinity toward the target protein or DNA molecule, allowing for easy binding with the analyte. SiC QDs are fabricated by a one-pot hydrothermal method [45]. The carboxylic group was activated by Ethyl-3-(3-dimethylaminopropyl) carbodiimide hydrochloride (EDC) and N-hydroxysuccinimide (NHS), with DNA added and incubated overnight. The mechanism to detect *P. mirabilis* based on fluorescence aptasensor assay is shown in Figure 1. EDC/NHS coupling leads to a condensation reaction between the carboxyl groups on the surface of SiC QDs and amino-modified DNA, which forms a fluorescence aptasensor. The aptamer with specific affinity provides connections between SiC QDs and *P. mirabilis*, as it can be bounded to the target bacteria via acting on the surface-associated proteins of *P. mirabilis*. This causes SiC QDs and *P. mirabilis* to form a complex, with the interaction of electron transfer from DNA-SiC QDs to the complex by the charges of both cellular proteins and DNA-SiC QDs. Such interaction is responsible for the fluorescence quenching of SiC QDs, which achieves quantitative testing of the *P. mirabilis* by the fluorescence change in the aptasensor [25].

**Figure 2:**
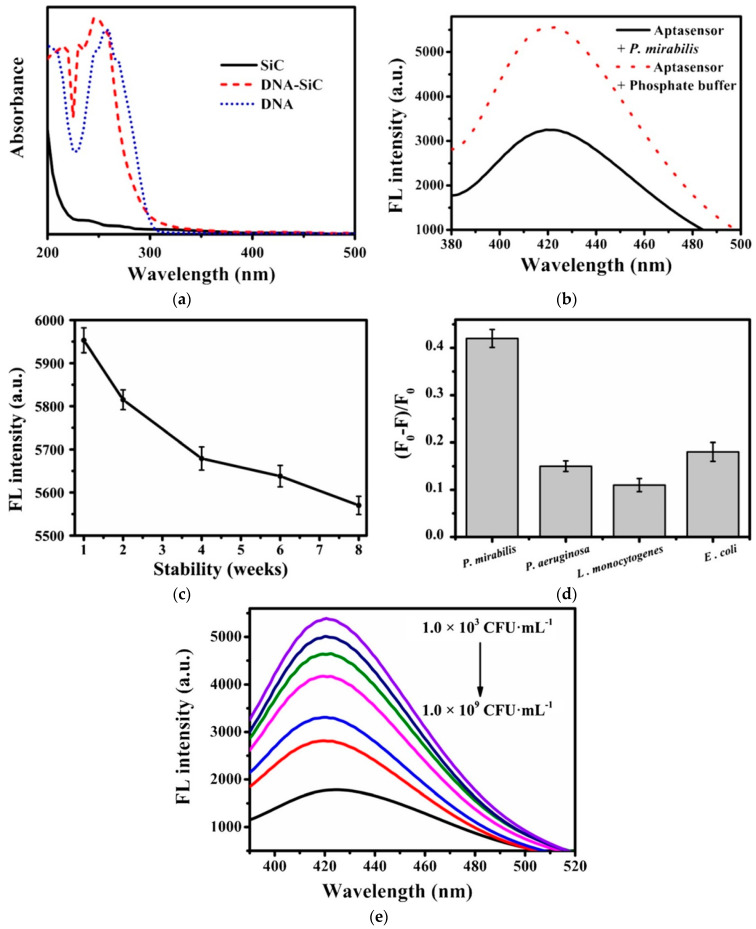
(**a**) UV-VIS spectra of SiC, DNA, and DNA-SiC QDs. (**b**) Fluorescent spectra of DNA-SiC QDs in the absence and presence of *P. mirabilis* with 1 × 10^7^ CFU mL^−1^. (**c**) The stability of the fluorescence intensity from SiC QDs in 8 weeks. (**d**) The selectivity of the aptasensor based on fluorescence intensity at 420 nm in the absence (F_0_) and presence (F) of bacteria (at 1.0 × 10^7^ CFU mL^−1^) including *P. mirabilis*, *P. aeruginosa, L. monocytogenes*, *E. coli*. (**e**) Fluorescence spectra of DNA-SiC QDs in the presence of *P. mirabilis* (10^3^–10^9^ CFU mL^−1^). Reprinted with permission from Ref. [25]. 2020, Microchimica Acta.

DNA sensing was performed by adding and incubating different concentrations of the target DNA (*P. mirabilis*), and then recording fluorescence spectroscopy. From the UV–visible absorption spectrum shown in Figure 2a, it can be seen that UV absorption of the aptamer was at 260 nm, while the peak shifted to 245 nm for the SiC-DNA interface by the successful binding of DNA with SiC QDs [45]. The significant shift in fluorescence spectrum before and after adding the target DNA in Figure 2b confirmed the sensitivity of the SiC QDs biosensor. The stability, selectivity, and sensitivity tests of the sensor were also performed. For stability testing, the SiC QDs were stored at 4 °C and then the fluorescence spectroscopy was measured each week. Figure 2c shows that after eight weeks of storage, the intensity of the SiC QD sensor reduced from 5953 a.u. to 5570 a.u., representing a 6.43% reduction. To test the selectivity, an experiment was performed under a similar testing condition by using different types of non-target DNA such as *P. aeruginosa*, *E. coli*, and *L. monocytogenes*. The result depicted in Figure 2d demonstrated that the sensitivity to non-target DNA was much lower than to target DNA. A quantitative analysis of the target DNA was performed by measuring different concentrations of *P. mirabilis* ranging from 10^3^ to 10^9^ CFU mL^−1^. The resulting fluorescent spectra are shown in Figure 2e where it can be observed that the fluorescent intensity decreases with an increase in DNA concentration. This work [25] demonstrated higher sensitivity than other methods [55,56,57] reported. This research work has successfully proved the feasibility of using a SiC QD-based biosensor for DNA-sensing applications. A similar type of DNA sensor was reported in [18] which proposed a method of stabilizing SiC nanoparticle’s surface by bacteria-targeting-bovine serum albumin (BSA) to detect DNA of *S. salivarius* found in human saliva.

### 2.2. SiC Nanoparticle-Modified Electrode for DNA Sensing

**Figure 3:**
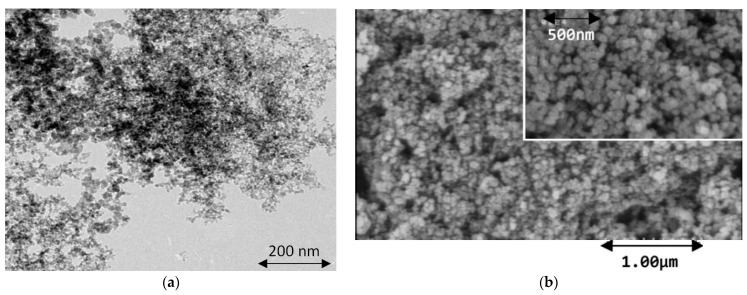
(**a**) TEM image of SiC powder, and (**b**) FE-SEM image of the SiCNP/GCE surface. The scale bar in (**a**) is 200 nm. Reprinted with permission from Ref. [1]. 2011, Biosensors and Bioelectronics.

In addition to desirable optical properties, SiC nanoparticles also have favorable electrical and electrochemical properties that can be utilized as an oxidation–reduction indicator in potentiometric titrations [58]. SiC nanoparticles were used to modify the surface of a glassy carbon (GC) electrode, with the resulting electrode device evaluated in the electrocatalytic detection of biomolecules [59]. In this study, SiC nanoparticles with a grain size of 20~40 nm were used to modify the surface of a glassy carbon electrode (GCE), followed by repetitive potential cycling applied to the device to improve the reproducibility and sensitivity of the electrode [1]. The surface morphology of the GCE modified with SiC nanoparticles was imaged via field-emission scanning electron microscopy (FE-SEM) as shown in Figure 3. The oxidation behaviors of purine (guanine and adenine) and pyrimidine (thymine and cytosine) bases in phosphate-buffered solution (PBS) were explored through cyclic voltammetry (CV) and differential pulsed voltammetry (DPV) testing.

**Figure 4:**
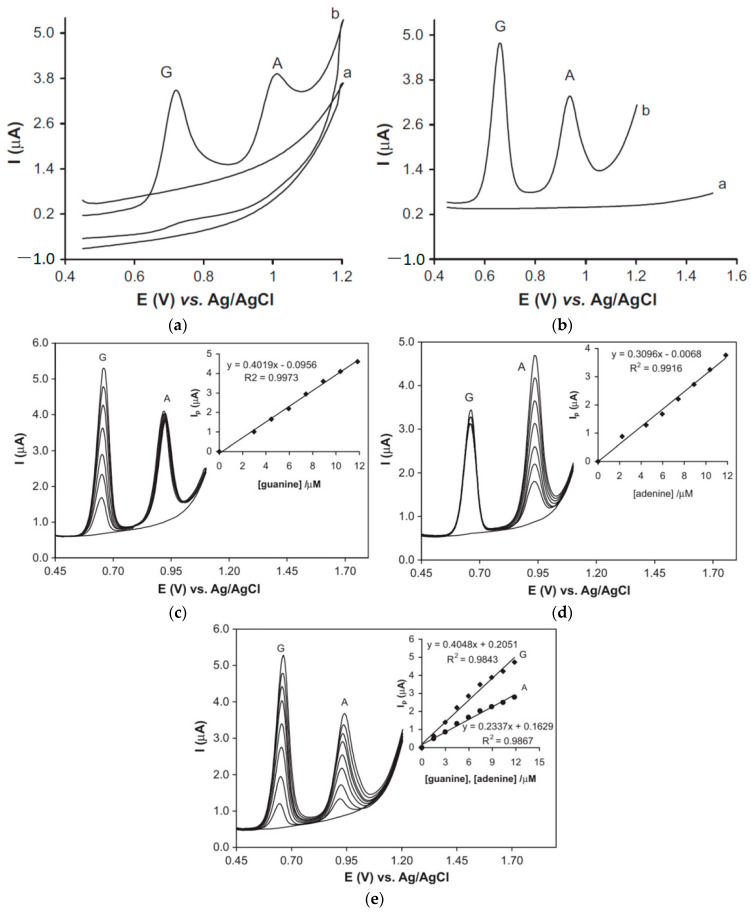
Successive cyclic voltammetry (scan rate: 100 mV/s) test results: (**a**) differential pulse voltammetrics (scan rate: 20 mV/s and pulse amplitude 50 mV); (**b**) in 0.1 M pH 7.4 PBS containing 12.0 mol L^−1^ guanine and 12.0 mol L^−1^ adenine at bare GCE (denoted as “a”) and modified SiCNP/GCE (denoted as “b”). (**c**) DPVs of various concentrations of guanine (G) from 3 µM to 12 µM in 4.85 µM adenine (A) solution, (**d**) various concentrations of adenine from 3 µM to 12 µM in 4.85 µM guanine solution, and (**e**) simultaneous determination of guanine and adenine from 1.8 to 1.2 µM. Inset: plots *I_p_* vs. concentrations. Reprinted with permission from Ref. [1]. 2011, Biosensors and Bioelectronics.

The CV and DPV curves for guanine and adenine using modified and unmodified GC electrodes are compared in Figure 4a,b, demonstrating that the unmodified surface had a negligible oxidation peak while the modified GC electrodes displayed irreversible oxidation processes. The oxidation peak for modified GC electrodes was found to be 0.64 V and 0.95 V for DNA bases guanine and adenine, respectively. Similar results were also obtained for thymine and cytosine. The DPV method was employed to establish the detection limit of the SiC nanoparticle-modified glass carbon electrode by testing various concentrations of guanine, adenine, thymine, and cytosine in PBS. Detection limits for guanine, adenine, thymine, and cytosine we found to be 0.015, 0.015, 0.14, and 0.14 mol L^−1^ (with S/N = 3 criteria), respectively. The sensor demonstrated high sensitivity, the ability to detect a wide range of DNA concentrations, and a low limit of detection. The independence of electrochemical signals between adenine and guanine, as well as between thymine and cytosine, was observed by conducting DPV tests with a fixed concentration of one base and a variable concentration of the other. The resulting response curves for guanine and adenine are shown in Figure 4c–e. The reported SiC nanoparticle-modified glass carbon electrode displayed excellent electrocatalytic oxidation activity toward DNA bases, with a wide range of sensitivity, selectivity, stability, and a negligible amount of interference with other biomolecules without the use of any specific reagents or preparation methods.

### 2.3. SiC Nanopillars for DNA Sensing

**Figure 5:**
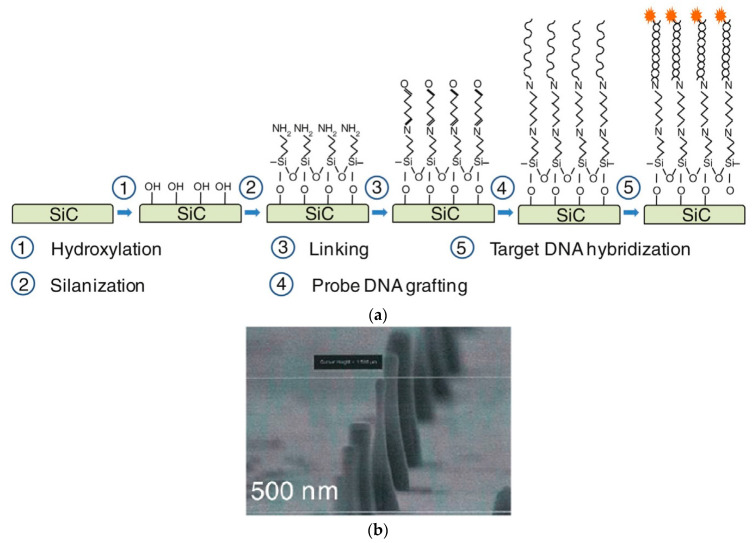
(**a**) Flowchart of the chemical process for DNA hybridization on a SiC surface. (**b**) SEM image of SiC nanopillar. Reprinted with permission from Ref. [23]. 2016, Elsevier.

One-dimensional nanostructures such as nanopillars have a unique advantage in sensing applications due to their high surface-to-volume ratio. SiC nanopillars combine the excellent semiconducting and biocompatibility properties of SiC with the exceptional sensing capabilities of nanopillars, resulting in a promising platform for DNA sensing. The first SiC-based nanopillar DNA biosensor [10] was fabricated on 6H-SiC and 4H-SiC wafers by a SF_6_/O_2_ plasma-etching process. The nanopillars had a height of 1.6 µm, diameter of 800 nm, density of 1020 pillars/cm^2^, and a 5 µm pitch. After fabrication, the SiC nanopillars were functionalized by a DNA grafting and hybridization process via chemical binding with aminopropyltriethoxysilane (APTES) and glutaraldehyde (GA) reagents in ethanol and water, respectively [23]; the functionalization process is shown in Figure 5.

**Figure 6:**
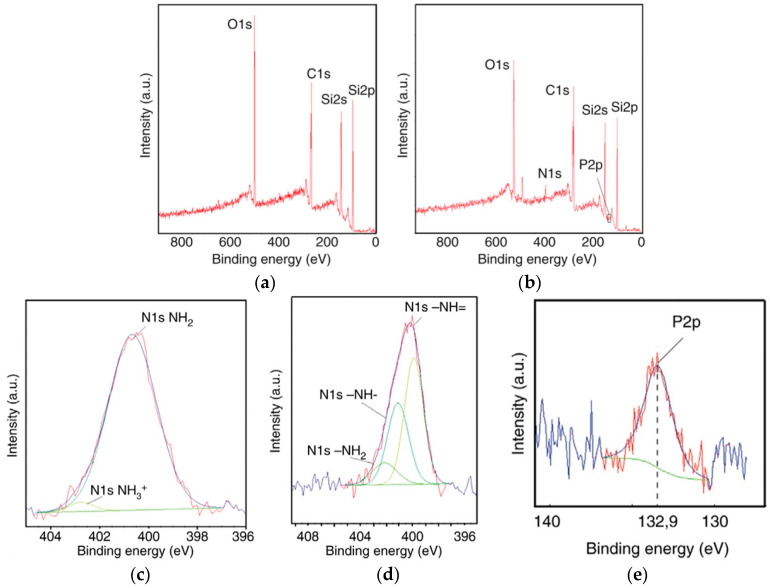
XPS surveys of (**a**) bare SiC wafer, (**b**) biomodified SiC sample with hybridized DNA, (**c**) N1s in the case of silanized SiC surface, (**d**) N1s in the case of hybridized DNA on SiC surface, and (**e**) P2p in the case of hybridized DNA on the SiC surface. Reprinted with permission from Ref. [23]. 2016, Elsevier.

The functionalized surfaces were characterized by X-ray photoelectron spectroscopy (XPS), fluorescence microscopy, and wettability measurement. Figure 6 shows the XPS measurement curves. The XPS data in Figure 6a show bare SiC with the peaks of Si, carbon (C), and oxygen (O) atoms. Figure 6b presents the XPS data after the hybridization process where additional peaks corresponding to nitrogen (N) and phosphorous (P) atoms appeared. Figure 6c presents the XPS data after the salinization process. In this figure, an additional peak corresponding to N is observed, which can be attributed to the presence of -NH_2_ and NH^3+^ molecules. Figure 6d presents the three core level peaks observed after DNA hybridization, related to -N=, -NH-, and -NH_2_. The presence of these peaks indicates a charge transfer mechanism between phosphorous (P2p) and nitrogen (N1s). In Figure 6d, the distance between the -N= and -NH- peaks is measured to be 1.22 eV, while the distance between the -NH- and -NH_2_ peaks is measured to be 1.04 eV. After DNA grafting, an extra P2p peak corresponding to the phosphate group of the DNA molecule appeared at a binding energy of 132 eV, as shown in Figure 6e. These measurements provide evidence for a DNA-coated surface [60]. These peaks after each process step confirmed the DNA grafting and hybridization process which is in agreement with the other literature [60,61]. It was confirmed from XPS measurement results that DNA was hybridized on the SiC nanopillars. This result opened up an opportunity for the future study of SiC-based nano-bio-FET [10].

### 2.4. SiC Nanowires for Optical DNA Sensor

**Figure 7:**
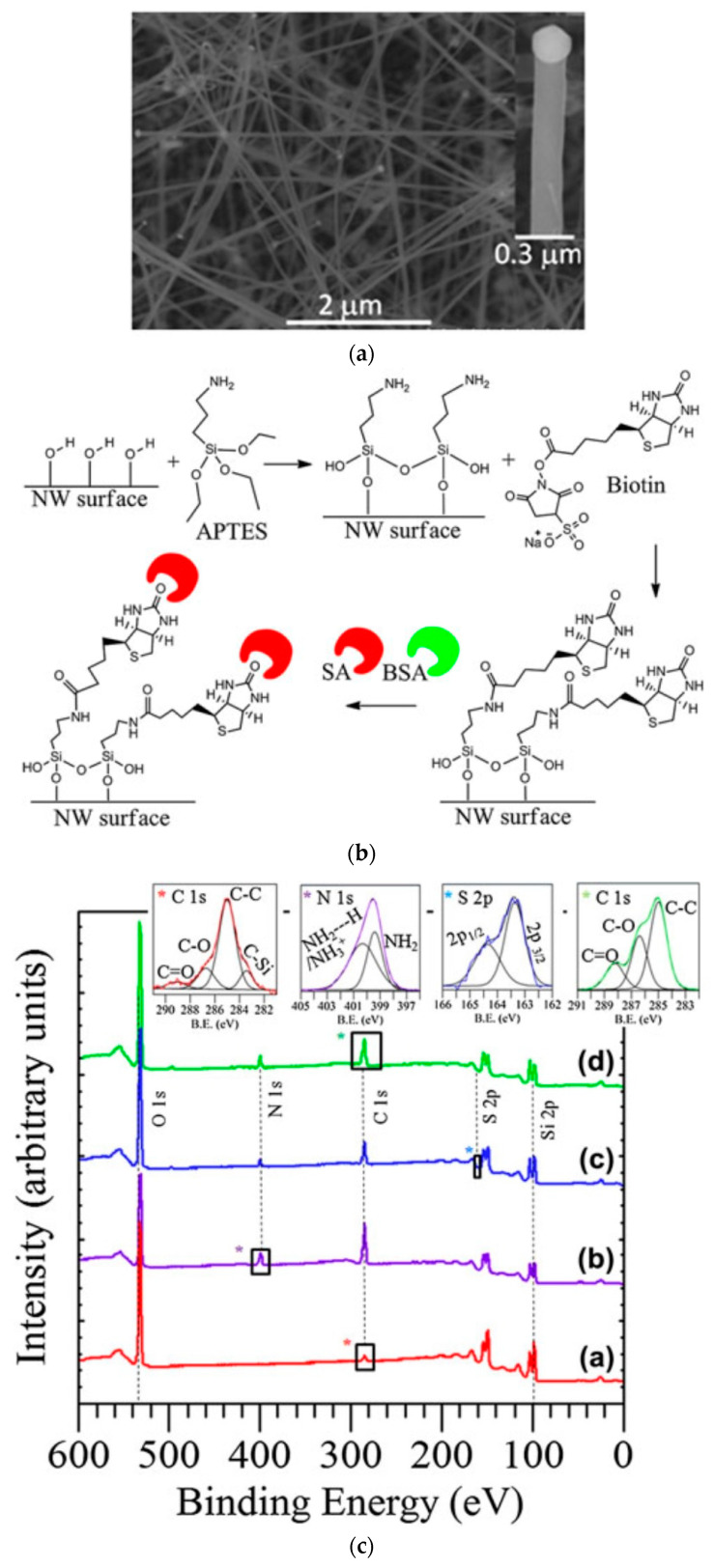
(**a**) FESEM image of 3C-SiC nanowires grown on a 4H-SiC (0001) substrate with individual nanowire tips shown in the inset. (**b**) Streptavidin (SA) binding and inhibition of Bovine Serum Albumin (BSA) binding to biotinylated nanowires. (**c**) XPS spectra of SiC nanowires. Curve a: as-grown; curve b: APTES-coated; curve c: biotinylated; and curve d: SA immobilized. The inset, from left to right, shows the C1s peaks from the as-grown SiC nanowires, the N1s peaks from NH_3_^+1^/NH_2_—H and NH_2_ after APTES functionalization, the S2p peaks after biotinylation, and the C1s signal related to SA conjugation. The different colors of * correspond to the color of the curves. Reprinted with permission from Ref. [19]. 2013, Journal of Materials Research.

SiC nanowires have been reported as a promising platform for optical DNA sensing by leveraging the optical properties of DNA [19]. In this paper, SiC nanowires were grown by using a chemical vapor deposition technique that employed a vapor–liquid–solid (VPS) mechanism on a 3C-SiC substrate. The SiC nanowires were reported to be 5~15 µm in length, with a diameter of 80–200 nm, as shown in Figure 7a. After growing the nanowires, a solution-based functionalization method was developed to chronologically immobilize 3-aminopropyltriethoxysilane (APTES), biotin, and streptavidin (SA) reagents on the nanowires, as shown in Figure 7b. After grafting DNA onto the surface of the nanowires, they were characterized by XPS, high-resolution transmission electron microscopy (HRTEM), and atomic force microscopy (AFM). The XPS spectrum after each functionalization step is shown in Figure 7c. The insets of the XPS spectrum from upper left to right correspond to the C1s peak from the as-grown SiC (curve a), the N1s peak from the functionalization reagent APTES (curve b), the S2p peak from the biotin (curve c), and the C1s peak from the SA reagent (curve d).

**Figure 8:**
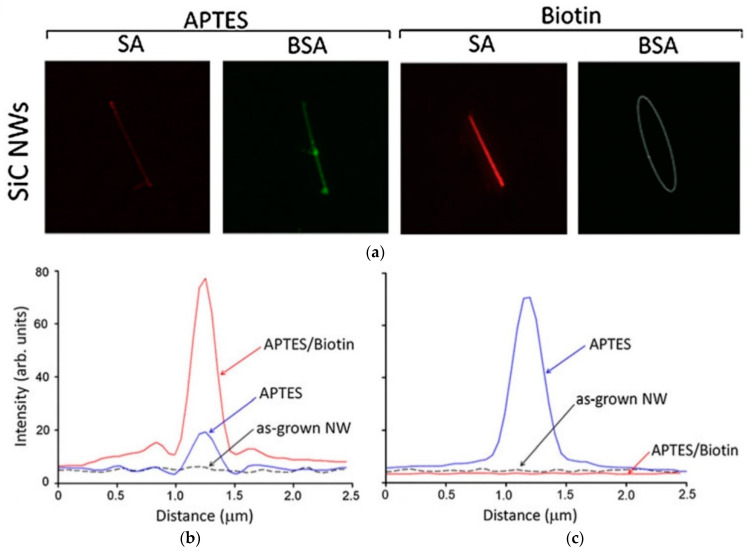
(**a**) Fluorescence microscopy images of APTES-coated and biotinylated SiC nanowires after exposure to the SA/BSA mixture. The same nanowire on APTES-coated samples and the same biotinylated nanowire on biotinylated sample is imaged by the red (SA) and green (BSA) fluorescence, respectively. The dashed oval on biotinylated sample marks the locations of nonfluorescent nanowires, confirming the absence of nonspecific BSA attachment. Fluorescence intensity of (**b**) SA and (**c**) BSA proteins from as grown, APTES-functionalized, and biotinylated SiC nanowires after exposure to the SA/BSA mixture. Reprinted with permission from Ref. [19]. 2013, Journal of Materials Research.

In order to graft the DNA, the functionalized surface of the SiC nanowires was exposed to a SA/bovine serum albumin (BSA) mixture. Followed by fluorescence microscopy to measure the efficacy of the DNA-grafting process. Fluorescence microscopic imaging and intensity profiles are shown in Figure 8. Figure 8a shows that the SA/BSA mixture did not attach to untreated SiC nanowires. This is due to the negatively charged native oxide on the nanowire surface, which repels the negatively charged BSA molecule [62]. In contrast, the SA/BSA mixture is strongly attached to APT-ES-treated SiC nanowires. This is due to the strong electrostatic attraction between the NH_3_^+^/NH_2_-H molecules of APTES and the negatively charged SA/BSA mixture [62], which is confirmed by the bright red-colored fluorescence image in Figure 8a. The fluorescence intensity profile is shown in Figure 8b,c, with the dotted line representing the as-grown SiC surface, which shows no affinity between the bare SiC and SA or BSA. On the other hand, the red line representing biotinylated SiC nanowires showed a significant intensity peak with SA but no peak with BSA. This suggests that SA has an affinity towards biotin, while BSA has no such affinity. The blue line in the figure represents the interaction of APTES-functionalized SiC nanowires with SA and BSA. This line showed a significant intensity peak for both SA and BSA, indicating that both proteins have a strong and specific affinity towards APTES-functionalized SiC nanowires.

### 2.5. SiC Nanowire-FETs for DNA Detection

Nanowire field effect transistors (nanowire-FETs) are one of the promising bio-sensing techniques and have rigorously been investigated [2,20,21,23,24,46,47]. These devices offer unique advantages, such as real-time and label-free detection [63] and high sensitivities for biomolecules such as DNA, RNA, proteins, and ions at femtomolar levels [5,64,65]. Since biomolecules such as DNA are electrically charged molecules, their adsorption on the surface of nanowire FETs can significantly change the transfer characteristics of the FET due to the high surface/volume ratio of the nanowire, which increases the sensitivity of the device [63]. SiC can be an ideal material for nanowire FET-based biosensors due to its favorable properties, as discussed in the earlier section, with various designs for DNA sensors reported in the literature [2,20,21,23,24,46].

To the fabrication of SiC nanowire-FET DNA sensors, highly doped n-type SiC nanowires were grown through a vapor–liquid–solid (VLS) process [66]. After the growth process, SiC nanowires were dispersed onto the substrate and then patterned using e-beam lithography to create micro-contacts. The micro-contacts were formed by depositing Ni and Au, which were subsequently lifted off to create the source and drain connections, respectively. This completed the FET structure [20,67]. After device fabrication, the transistor characteristics of the SiC nanowire-FETs were determined [68] using the backside of the wafer as the gate contact.

**Figure 9:**
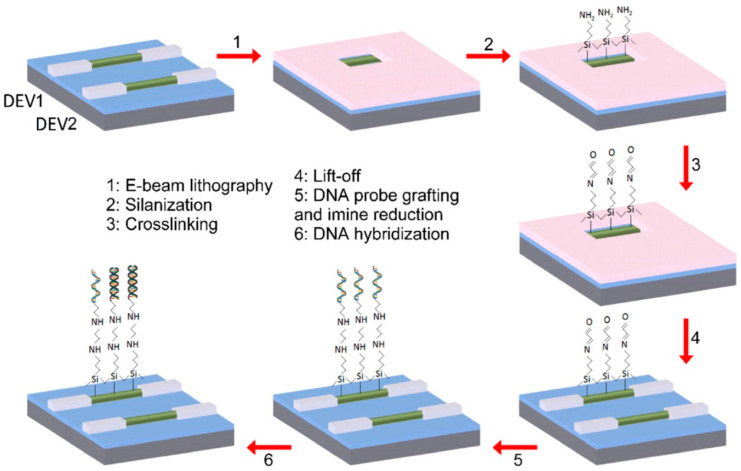
A flow-chart of the localized functionalization process for DNA grafting and hybridization on SiC nanowire nanosensor (DEV1). The reference (DEV2) is kept un-functionalized. Reprinted with permission from Ref. [63]. 2022, Microelectronic Engineering.

The SiC nanowire-FETs were functionalized by DNA grafting and hybridization via covalent coupling using an aminoterminated organosilane [21] reagent, following the steps shown in Figure 9. The functionalized surface was characterized using XPS, fluorescence microscopy and AFM [69,70]. The DNA-sensing tests were performed using a configuration that consisted of two SiC nanowire-FETs on the same chip; one sensor was functionalized with DNA, while the other was not functionalized and served as the reference device [63]. The test was performed in a dry state, as dry state measurement enhances the sensitivity to negatively charged molecules on the n-doped nanowires [71], and also mitigates the microfluidic and ionic effects [63].

**Figure 10:**
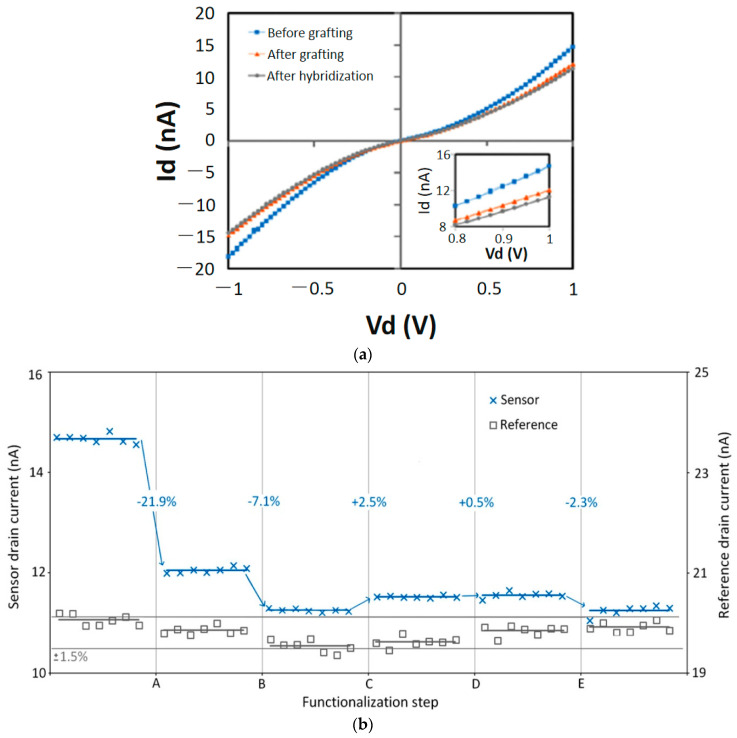
(**a**) *I_d_* vs. *V_d_* characteristics of a SiC nanowire at *V*_g_ = 0 V before DNA probe grafting (initial state), after DNA probe grafting and after hybridization with DNA targets. (**b**) The drain current evolution of the sensor (DEV1, functionalized) and reference sample (DEV2, un-functionalized) after different functionalization steps: silane and glutaraldehyde covalent bonding (initial step), DNA probe grafting (step A), complementary hybridization (step B), dehybridization (step C), non-complementary hybridization (step D), and complementary re-hybridization (step E). The percentages represent the variation of the mean current between two successive steps. Reprinted with permission from Ref. [63]. 2022, Microelectronic Engineering.

The SiC nanowire FETs were characterized before and after DNA grafting as well as after DNA hybridization, with the *I_d_* vs. *V_d_* curves shown in Figure 10a. The current of functionalized device (DEV1) was measured when a voltage between −1 V to +1 V was applied before DNA probe grafting (initial), after DNA probe grafting (step B), and finally after DNA hybridization (step 2). It shows that the current reduced from 14.72 nA before DNA grafting to 12 nA after DNA probe grafting. After hybridization, the current value was further reduced to 11.25 nA. The continuous decrease in current is a result of the n-type SiC channel being reduced by the negatively-charged DNA molecules at each step [20]. The *I_d_* vs. *V_d_* characteristic curves from the SiC nanowire-FET DNA sensor and the reference device were measured simultaneously for comparison after different surface modification steps: initial state—silane and glutaraldehyde covalent bonding, A—DNA grafting, B—hybridization, C—de-hybridization, D—hybridization with DNA target probe, and E—rehybridization with complementary target DNA. The results are shown in Figure 10b. The current measured from the SiC nanowire-FET DNA sensor was reduced by 21.9% after DNA grafting and further reduced by 7.1% after DNA hybridization due to the field effect being impacted by the negatively charged DNA molecules, while the measured current change from the reference device was very small (<±0.6%) [63]. This result agrees with findings reported from Si-based nanowire-FET DNA sensors [72,73]. Similar results have been also reported by [2,20,21,23,24,46], further supporting the sensitivity of SiC nanowire-FETs for DNA sensing.

### 2.6. Nanocrystalline 3C-SiC Electrode for DNA Detection

**Figure 11:**
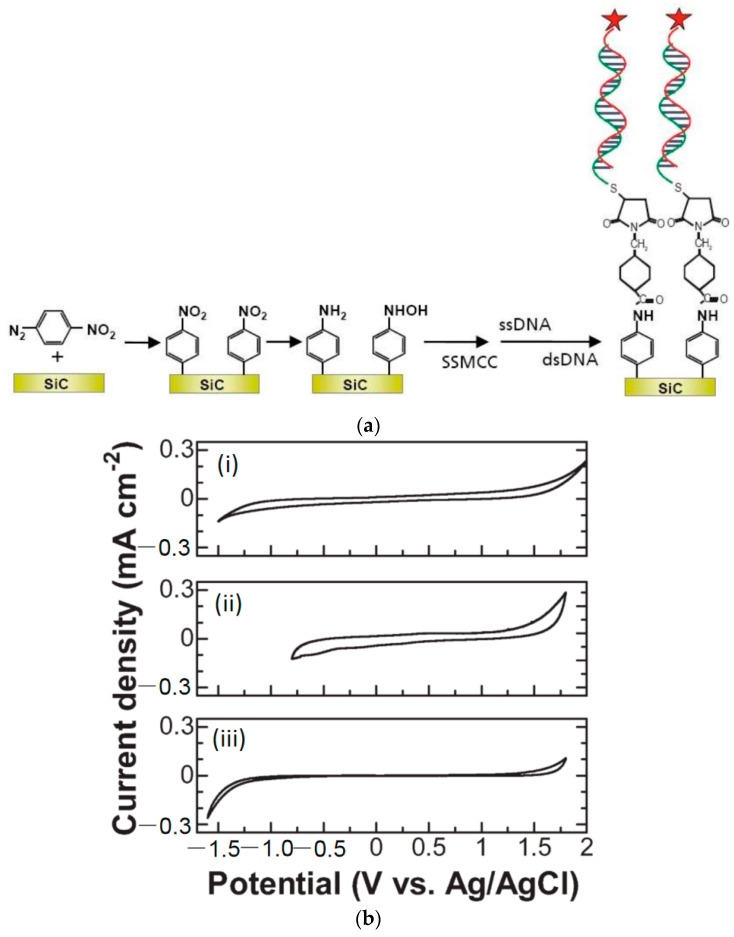
(**a**) Schematic of bio-functionalization of SiC with DNA. SSMCC: the cross-linker of sulphosuccinimidyl-4-(N-maleimidomethyl) cyclohexane-1-carboxylate. (**b**) Cyclic voltammograms from (i) nanocrystalline 3C-SiC electrode, (ii) glassy carbon electrode, and (iii) boron-doped (5 × 10^23^ cm^−3^) diamond electrode in 0.1 M H_2_SO_4_ at a scan rate of 100 mV/s. Reprinted with permission from Ref. [22]. 2011, Analytical Chemistry.

N-type and p-type nanocrystalline SiC can be grown [22] and has been also reported as an excellent candidate for DNA sensing and biosensing applications [22,41,48,49]. As reported in [50], a 3C-SiC nanocrystalline film was deposited on top of a p-type Si (100) substrate as an electrode and functionalized with diazonium salt. During DNA sensing, the DNA molecules were covalently bonded to the nitrophenyl-linker molecule as shown in Figure 11a. Cyclic voltammetry (CV) measurements were used to characterize the electrochemical potential window and background noise of a nanocrystalline 3C-SiC electrode without surface functionalization, to evaluate its potential for electrochemistry. The results shown in Figure 11b indicate that 3C-SiC has a wider potential window (3.0 V) compared to an electrode made from glassy carbon (2.0 V), and 3 to 5 times lower background noise.

**Figure 12:**
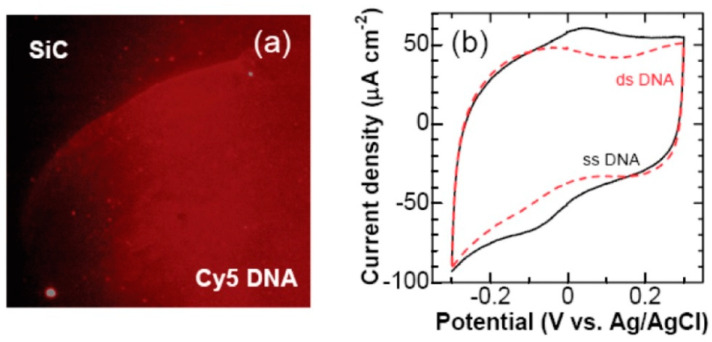
(**a**) Fluorescence microscopic image of a ds-DNA functionalized SiC electrode. (**b**) Cyclic voltammograms of ss-DNA (solid line) and ds-DNA (dashed line)-modified SiC electrode in a pH 7.4 PBS at a scan rate of 50 mV/s. Reprinted with permission from Ref. [22]. 2011, Analytical Chemistry.

After immobilization of the DNA on both the bare and functionalized SiC surfaces, fluorescence microscopy was applied to examine the adsorption of DNA on both surfaces. In the fluorescence image of the bare SiC surface, as shown in the upper left corner of Figure 12a, no red fluorophore emission was found, indicating that no adsorption of DNA occurred on the bare SiC surface. On the contrary, in the fluorescence image of the functionalized SiC surface, as shown in the lower right corner of Figure 12a, a high-intensity level of red fluorophore was found, indicating DNA adsorption had occurred on the functionalized SiC surface. Furthermore, cyclic voltammetry was performed to evaluate the sensitivity of the functionalized SiC towards the target DNA molecule. In the voltammogram shown in Figure 12b, the redox wave was observed to decrease for double-stranded (ds) DNA or hybridized DNA, as indicated by the red dashed curve, in comparison to the single-stranded (ss) DNA, represented by the solid line. This is because the hybridized DNA prevents electron transfer from the SiC electrode surface to the solution and therefore reduces the redox current. Such a decrease in redox current indicates the presence of the target DNA on the functionalized SiC surface and demonstrates the sensitivity of the sensor towards DNA molecules [22].

## 3. Future Prospects and Challenges

Various forms of SiC devices for DNA sensing, such as nanoparticle or quantum dots, nanowire, nanopillar, planar electrodes, among others, have been reported with promising results. The SiC quantum dot-modified aptasensor demonstrated efficiency in the selective determination of *P. mirabilis* and was capable of evaluating targeted DNA quantity in pure milk [25]. Glassy carbon electrodes modified with SiC nanoparticles showed feasibility for DNA sensing without the need for a pretreatment procedure [1]. The results reported by the SiC nanowire/nanopillar sensors opened a path to SiC nanowire FET biosensors [10,21]. SiC nanowire FET demonstrated excellent potential for sensing DNA molecules in a resistive configuration with high sensitivity, selectivity, reproducibility, and stability [20]. Additionally, planar SiC electrodes have shown encouraging results for electrochemical sensing of DNA molecules. A summary of these technologies is listed in Table 2. 

It is noted that challenges still remain within these sensing technologies. Regarding the SiC quantum dot-modified aptasensor, interference from other molecules in the solution was observed, so a reference solution containing the exact DNA of interest was required for accurate testing [25]. For SiC nanowire FET sensors, their performance is influenced by stacking faults and unintentional doping, as well as the chemical characteristics of the functionalization layer and the length of the DNA chains. Additionally, the humidity level can also impact the field effect of the sensor. [20,63]. For planar SiC electrodes, the potential window, noise current, and voltammetry reactivity must be optimized [22]. Finally, for all these sensing technologies it is critical to evaluate the influence of non-specific binding molecules, humidity, temperature, and other factors on the performance of the sensor [23,26].

## 4. Conclusions

This article provides a review of SiC-based DNA-sensing technologies currently reported in the literature. These technologies utilize various forms of SiC, including nanoparticles and quantum dots, nanowires, nanopillars, and planar electrodes, which employ optical, chemical, electrical, and electrochemical-sensing methods. The SiC quantum dot-based fluorescent aptasensor has shown promising results in the quantitative analysis and sensing of target bacteria through optical sensing methods, offering ease, speed, and long-term stability. The glassy carbon electrode modified with SiC nanoparticle exhibited great potential for electrochemical sensing of the four DNA bases with high sensitivity, selectivity, simplicity, speed, and stability. Regarding SiC nanopillars, the successful hybridization and grafting of DNA molecules on top of and around the nanopillars was verified by chemical analysis using XPS and fluorescence microscopy, indicating the promising potential of SiC nanopillars in DNA sensing applications. In addition, functionalized SiC nanowires demonstrated an affinity for SA and BSA molecules as confirmed by XPS, HRTEM, and fluorescence microscopy, which further illustrates the future potential of SiC nanowires for biomolecule and DNA sensing applications. Another sensing technology reviewed was SiC nanowire-FETs, which exhibited a consistent decline in mean drain current during each stage of DNA probe grafting and target DNA hybridization, demonstrating the high sensitivity of the device. The nanocrystalline planar SiC electrode also demonstrated outstanding electrochemical characteristics in DNA sensing during cyclic voltammetry and differential pulse voltammetry measurements. In summary, SiC-based technologies for DNA sensing have shown promising results in detecting and analyzing DNA molecules using various sensing methods. These technologies have the potential to offer high sensitivity, selectivity, simplicity, speed, and stability, while leveraging the favorable material properties of SiC such as its biocompatibility and chemical inertness, making them suitable for DNA-sensing applications. However, while much of the fundamental groundwork has been laid, further research is needed to optimize and improve the performance of these technologies.

## Figures and Tables

**Table 1:** Structural and material properties of 3C, 4H, and 6H-SiC polytypes.

Material Properties	6H-SiC	3C-SiC	4H-SiC
Crystal structure	Hexagonal	Zinc-blende	Hexagonal
Band gap *E_g_* (eV, RT *)	2.9	2.2	3.2
Melting point (K)	>2000 Sublime	>2000 Sublime	>2000 Sublime
Physical stability	Excellent	Excellent	Excellent
Electron mobility *m_n_* (RT, cm^2^/Vs) **	||c-axis: 60 ˔c-axis: ~400	750~1000	||c-axis: 800 ˔c-axis: 950
Hole mobility *m_p_* (RT, cm^2^/Vs) **	90	40	115
Breakdown field *E_Br_* (MV/cm)	||c-axis: 3~3.2	>1.5	||c-axis: 3~3.5
Saturation electron velocity *v_sat_* (10^7^ cm/s)	2	2.5	2
Relative dielectric constant *e_r_*	9.7	9.7	9.7
Thermal conductivity *k* (W/cm-K)	3.3~3.9	3.3~3.9	3.3~3.9 4.9 (S.I.) ***
RT intrinsic carrier concentration (cm^−3^)	~10^−5^	~10	~10^−7^

* RT: room temperature, ** Low doping density (~10^16^ cm^−3^), *** S.I. = Semi-insulating 4H-SiC wafers, || parallel, ˔ normal.

**Table 2:** A summary of SiC technologies for DNA sensing.

Technology	Sensitivity	Detection Limit	Response Time	Detection Method	Accuracy	Linear Detection Range
Aptamer-modified SiC nanoparticle and quantum dot aptasensor	X	526 CFU/mL	35 min	Fluorescence	87.6–104.3%	10^3^–10^8^ CFU/mL
SiC nanoparticle-modified glassy carbon electrode	Guanine: 0.3877 (µA/µM)	0.015 (µM)	X	Voltammetry, Differential Pulse Voltammetry	X	0.1–12 (µM)
	Adenine: 0.3289 (µA/µM)	0.015 (µM)	X		X	0.1–12 (µM)
	Cytosine: 0.0175 (µA/µM)	0.14 (µM)	X		X	1.2–136 (µM)
	Thymine: 0.0499 (µA/µM)	0.14 (µM)	X		X	1.2–136 (µM)
SiC nanopillars	X	X	X	XPS, Fluorescence	X	X
SiC nanowire optical sensor	X	X	X	XPS, Fluorescence, AFM, HRTEM	X	X
SiC nanowire FETs	X	X	X	Electrical	X	X
Nanocrystalline 3C-SiC electrode	X	X	X	Voltammetry, XPS, Fluroscence	X	X

## Data Availability

No new data were created or analyzed in this study. Data sharing is not applicable to this article.
